# Research Reality in Neuroradiology: a Survey Analysis at German University Hospitals

**DOI:** 10.1007/s00062-025-01608-2

**Published:** 2026-01-28

**Authors:** Béatrice Maxime von Tresckow, Katharina Schregel, Sarah Schlaeger, Daniel P. O. Kaiser, Roland Schwab, Daniel Weiss, David Zopfs, Elke Hattingen, Ansgar Berlis, Peter Schramm, Katharina J. Wenger

**Affiliations:** 1 Institute of Neuroradiology and Cooperative Brain Imaging Center, Goethe University and Goethe University Hospital, Frankfurt, Germany; 2 Department of Diagnostic and Interventional Radiology – Section Neuroradiology, Jena University Hospital, Jena, Germany; 3 Department of Diagnostic and Interventional Neuroradiology, School of Medicine and Health, TUM Klinikum Rechts der Isar, Technical University of Munich, Munich, Germany; 4 Department of Radiology, LMU University Hospital, LMU Munich, Munich, Germany; 5 Institute of Neuroradiology, Faculty of Medicine and University Hospital Carl Gustav Carus, Technische Universität Dresden, Dresden, Germany; 6 University Clinic for Neuroradiology, University Hospital Magdeburg, Magdeburg, Germany; 7 Research Campus STIMULATE, Otto-von-Guericke University Magdeburg, Magdeburg, Germany; 8 Department of Diagnostic and Interventional Radiology, University Düsseldorf, Medical Faculty, Düsseldorf, Germany; 9 Institute for Diagnostic and Interventional Radiology, University of Cologne, Faculty of Medicine and University Hospital Cologne, Cologne, Germany; 10 Department of Neuroradiology, University Hospital Augsburg, Augsburg, Germany; 11 Department of Neuroradiology, University Hospital Schleswig Holstein Campus Luebeck, Luebeck, Germany

**Keywords:** DGNR, Research satisfaction, Mentoring, Clinician scientist, Structural independence

## Abstract

**Purpose:**

This study aimed to provide a representative overview of the current research conditions in neuroradiology at German hospitals.

**Methods:**

In 2024, the German Society of Neuroradiology (DGNR) conducted an anonymous online survey targeting neuroradiology researchers working at German hospitals. Participants were recruited via targeted e-mail-outreach and professional social media channels. Data were collected using the Easyfeedback platform. A total of 60 individuals participated, of whom 33 completed the full 33-item questionnaire. Data were analyzed descriptively.

**Results:**

Among respondents who completed the survey, 50% reported being satisfied or very satisfied with their current research conditions. Respondents from non-independent departments expressed more dissatisfaction than those of independent institutes. Major challenges for researchers included insufficient research time, lack of funding, and limited infrastructure. Scientific work was most frequently conducted alongside clinical duties or during personal time, while only a minority of respondents reported receiving continuousely protected research time. Intramural university funding represented the most common funding source. Any type of structured research program was available at approximately half of the institutions. Mentoring opportunities within these programs were reported by a majority of respondents, with generally positive evaluations and corresponding higher satisfaction with research conditions.

**Conclusion:**

Structural independence, expanding protected research time, improving funding opportunities, and promoting structured research and mentoring programs appear essential to ensure sustainable academic development and innovation in the field.

## Introduction

In 1896, Hermann Welcker captured the first lateral skull radiograph—an endeavor that took one hour. Since then, neuroradiology has developed into a distinct and rapidly evolving specialty, significantly contributing to diagnostic accuracy, patient care, and clinical research. It is now recognized as an independent discipline in most German university hospitals, reflecting its growing clinical and academic importance [1].

Despite strong interest in research among radiologists, earlier surveys have documented widespread dissatisfaction with institutional support structures [2]. In particular, younger physicians report exhaustion, administrative overload, and a lack of structured academic training opportunities [3, 4].

According to data from the Federal Statistical Office (Destatis), physician density in Germany increased from 28 physicians per 10,000 inhabitants in 1991 to 45 physicians per 10,000 inhabitants in 2021 [5]. While an increase in the number of physicians combined with a reduction in working hours and an increase in pay might be expected to have a beneficial impact on physicians’ well-being [6], a 2023 study by the Deutsche Röntgengesellschaft (DRG) revealed that 76.7% of respondents experienced symptoms of burnout [7]. International comparisons further highlight systemic disparities: while in the United States there are 0.3 residents per attending, the average in Germany is 2.9—suggesting a considerable gap in faculty supervision and mentorship. Adequate staffing and mentoring are essential for high-quality research, teaching, and clinical performance [8].

In light of mounting economic and demographic pressures across the healthcare system, the Deutsche Gesellschaft der Neuroradiologie (DGNR), established in 1970, launched a national survey in 2024 to assess the state of research in neuroradiology at German university hospitals. The survey aimed to identify key structural and systemic challenges under those conditions, focusing specifically on research time allocation, funding mechanisms, mentoring practices, clinician-scientist pathways, and interdisciplinary collaboration.

## Methods

### Study Design and Participants

This study was designed as a descriptive, exploratory survey without a predefined hypothesis targeting researchers affiliated with neuroradiology institutes/departments at German hospitals. The DGNR identified potential participants through a systematic search of institutional websites, focusing on presented research groups. Researchers were contacted via email in three consecutive waves over a nine-day period. A total of 145 individuals were invited to participate, with instructions to forward the invitation within their departments to maximize outreach. As forwarding behaviour could not be tracked, the total number of additional recipients beyond these 145 direct addressees remains unknown. To complement direct recruitment, the study was also promoted through professional social media channels, particularly of the young members’ organization Junge Neuroradiologie (JuNRAD) [9], with the intention of also reaching younger researchers, assuming that social media are more frequently used by this group. However, the survey was not restricted to young members; all research-active neuroradiology researchers were eligible to participate, so that respondents represent a broader cross-section of the neuroradiology research community rather than exclusively junior members.

### Survey Implementation

The survey was administered anonymously using the Easyfeedback platform and remained open for two months, from October 28 to December 26, 2024.

### Questionnaire Development

The instrument consisted of 33 items covering both quantitative and qualitative data. Items used categorical response options; no formal Likert scale instrument was used. It was structured into the following thematic sections:

1. Structural organization of neuroradiology departments/institutes
2. Use of artificial intelligence (AI) in research
3. Availability of research programs and funding opportunities
4. Publication output and institutional support
5. Researcher satisfaction with existing research conditions

Questions were developed based on previously published surveys in related disciplines to enable a comparative analysis and were expert reviewed and pilot tested by the spokespeople of the young members’ organization JuNRAD [2, 10, 11].

### Data Analysis

Following survey completion, data were exported from Easyfeedback to Excel and analyzed descriptively. Results are reported as absolute numbers and relative frequencies. As response rates and answer options varied, denominators differ between items; sample sizes (*n*) are indicated for each table.

## Results

### Participant Characteristics

A total of 60 individuals responded to the survey, with 33 completing the full 33-item questionnaire and 27 providing partial responses (Table 1). To preserve anonymity, no individual demographic data such as age or sex were collected. Due to the high dropout rate, all available data per question were used, so the number of observations varies across items and decreases towards the end of the questionnaire. No formal reporting guideline (e.g., STROBE or CHERRIES) was applied to the design or reporting of this survey.

**Table 1:** Participant Characteristics

Participants		Percent (number/number of respondents to question)
	Total	100.00% (*n* = 60/60)
	Completed	55.00% (*n* = 33/60)
	Partially completed and prematurely terminated	45.00% (*n* = 27/60)

### Institutional Structure and Staffing

**Fig. 1:**
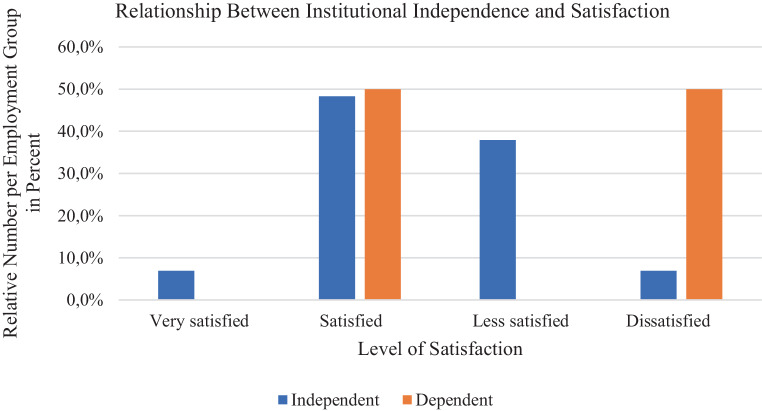
Satisfaction levels by institutional independence

Participants represented a diverse range of professional roles: institute directors 29.03%, (*n* = 18/62), section heads 24.19% (*n* = 15/62), clinical scientists 22.58% (*n* = 14/62), working group leaders 16.13% (*n* = 10/62), postdoctoral researchers 4.84% (*n* = 3/62), and doctoral students 3.23% (*n* = 2/62). No responses were received from medical scientists without clinical duties. Among respondents, 69.23% (*n* = 36/52) work in structurally independent neuroradiology institutes, while 30.77% (*n* = 16/52) belong to structurally subordinate units within general radiology. Dissatisfaction was lower in independent institutes, while half of the employees in non-independent structures reported to be dissatisfied (Fig. 1). In neuroradiology institutes with more than five employees, resident physicians 57.14% (*n* = 28/49) and senior attending physicians 53.06% (*n* = 26/49) dominate. The composition of the scientific working groups is diverse: in addition to Bachelor’s/Master’s students 17.82% (*n* = 18/101), there are doctoral students and post-doctoral students both from the medical discipline 28.71% (*n* = 29/101), as well as natural sciences 17.82% (*n* = 18/101) and 16.83% (*n* = 17/101) (Table 2).

**Table 2:** Institutional Structure and Staffing

Question/Analysis	Response options	Percent (number/number of respondents to question)
I work as a …	Medical scientist without clinical duties	0.00% (*n* = 0/62)
	Doctoral candidate	3.23% (*n* = 2/62)
	Postdoc	4.84% (*n* = 3/62)
	Group leader	16.13% (*n* = 10/62)
	Clinician Scientist	22.58% (*n* = 14/62)
	Section Head	24.19% (*n* = 15/62)
	Institute director	29.03% (*n* = 18/62)
What is the staff composition of your institute or department? (e.g., number of residents, senior physicians, etc.)	Number of attending physicians with special responsibilities (German: ”Funktionsoberärzte”)	< 5: 76.09% (*n* = 35/46)
		> 5: 8.70% (*n* = 4/46)
		No response: 15.22% (*n* = 7/46)
	Number of board-certified specialists (German: “Fachärzte”)	< 5: 61.70% (*n* = 29/47)
		> 5: 36.17% (*n* = 17/47)
		Not specified: 2.13% (*n* = 1/47)
	Number of senior attending physicians (German: “Oberärzte”)	< 5: 44.90% (*n* = 22/49)
		> 5: 53.06% (*n* = 26/49)
		Not specified: 2.04% (*n* = 1/49)
	Number of resident physicians (German: ”Assistenzärzte”)	< 5: 34.69% (*n* = 17/49)
		> 5: 57.14% (*n* = 28/49)
		Not specified: 8.16% (*n* = 4/49)
If you are part of a research group, please indicate the number of group members in each category.	Bachelor/Master students	17.82% (*n* = 18/101 reported group members)
	Medical doctoral candidates	28.71% (*n* = 29/101 reported group members)
	PhD students	17.82% (*n* = 18/101 reported group members)
	Postdoctoral Fellow (MD)	28.71% (*n* = 29/101 reported group members)
	Postdoctoral Fellow (non-MD)	16.83% (*n* = 17/101 reported group members)
Is neuroradiology at your institution an independent institute/department or a subdivision of general radiology (subordinate unit)?	Structurally independent	69.23% (*n* = 36/52)
	Department or a subdivision of general radiology (subordinate unit)	30.77% (*n* = 16/52)
Employee satisfaction was analyzed in relation to the institutional structure.	Very satisfied:	Independent: 6.90% (*n* = 2/29)
		Non-independent: 0.00% (*n* = 0/0)
	Satisfied:	Independent: 48.30% (*n* = 14/29)
		Non-independent: 50.00% (*n* = 2/4)
	Less satisfied:	Independent: 37.90% (*n* = 11/29)
		Non-independent: 0.00% (*n* = 0/0)
	Dissatisfied:	Independent: 6.90% (*n* = 2/29)
		Non-independent: 50.00% (*n* = 2/4)

### Research Focus and Time Allocation

The majority of respondents’ research focus is on clinically oriented research, with 55.22% (*n* = 37/67) of respondents, followed by basic experimental research 31.34% (*n* = 21/67) and epidemiological research 13.43% (*n* = 9/67). Within clinical research, neurovascular research 27.08% (*n* = 13/48) and general neuroimaging 25.00% (*n* = 12/48) dominate. Other areas include interventional neuroradiology 17.00% (*n* = 8/48), neuro-oncology 12.50% (*n* = 6/48), artificial intelligence 6.25% (*n* = 3/48), clinical neuroradiology and neurology 4.17% (*n* = 2/48) each, as well as psychosomatic/systemic influences 2.08% (*n* = 1/48). Scientific research is primarily conducted alongside clinical duties during regular working hours 39.39% (*n* = 26/66) or during leisure time 37.88% (*n* = 25/66). Only 22.73% (*n* = 15/66) of respondents profited from dedicated, protected research time. Among those with dedicated research time, the majority benefited from their research time on a daily basis 53.86% (*n* = 21/39), followed by weekly 25.64% (*n* = 10/39), hourly 12.82% (*n* = 5/39), and continuous full-time arrangements 7.69% (*n* = 3/39). The majority of respondents who reported arrangements for protected research time were fully granted that time, two even reported to be granted more than officially allocated. Only *n* = 6/24 respondents were not reliably granted their protected research time in practice (Table 3).

**Table 3:** Research Focus and Time Allocation

Question/Analysis	Possible answer	Percent (number/number of respondents to question)
When do you mainly carry out your scientific work?	During working hours in addition to clinical work	39.39% (*n* = 26/66)
	Leisure time	37.88% (*n* = 25/66)
	Research within the framework of an official research exemption	22.73% (*n* = 15/66)
If you are granted some sort of protected research time, what is the structure of it?	Allocated by full days	53.86% (*n* = 21/39)
	Allocated on a weekly basis	25.64% (*n* = 10/39)
	Allocated by hours	12.82% (*n* = 5/39)
	Full-time protected research	7.69% (*n* = 3/39)
If you have an official research exemption, what percentage of your total working time does this represent?	Officially allocated percentage	Percent for respondents does not apply here therefore median given: 25 (range 0-100); (*n* = 24)
	Actual percentage in practice	Percent of respondents does not apply here therefore median given: 26 (range 0-100); (*n* = 20)
Which scientific field does your research mainly focus on?	Clinically oriented research	55.22% (*n* = 37/67)
	Experimental basic research	31.34% (*n* = 21/67)
	Epidemiological research	13.43% (*n* = 9/67)
Subspecialties named within clinically oriented research (free text)	Neurovascular research	27.08% (*n* = 13/48)
	Neuroimaging/Imaging	25.00% (*n* = 12/48)
	Interventional neuroradiology	17.00% (*n* = 8/48)
	Neuro-oncology	12.50% (*n* = 6/48)
	Artificial intelligence	6.25% (*n* = 3/48)
	Clinical Neuroradiology	4.17% (*n* = 2/48)
	Neurology	4.17% (*n* = 2/48)
	Psychosomatic and systemic influences	2.08% (*n* = 1/48)
	N/A	2.08% (*n* = 1/48)

### Use of Artificial Intelligence in Research

**Fig. 2:**
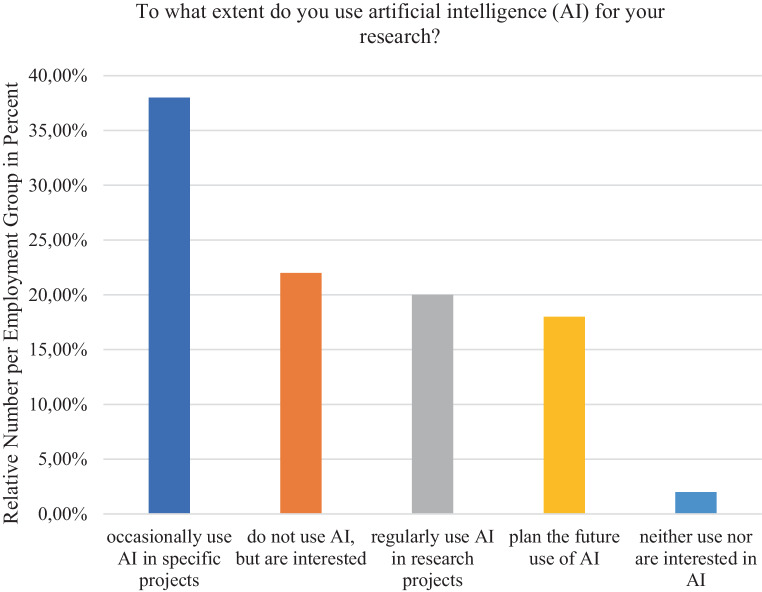
Extent of AI use in research

AI was actively used in research by a substantial proportion of participants. Specifically, 38.00% (*n* = 19/50) reported occasional use in project-based work, 20.00% (*n* = 10/50) used AI regularly, and 18.00% (*n* = 9/50) planned a future implementation. An additional 22.00% (*n* = 11/50) expressed interest without current use, while only 2.00% (*n* = 1/50) reported no use or interest (Fig. 2; Table 4).

**Table 4:** Use of Artificial Intelligence in Research

Question	Possible answer	Percent (number/number of respondents to question)
To what extent do you use artificial intelligence (AI) in your research?	Occasionally use AI in specific projects	38.00% (*n* = 19/50)
	Do not use AI, but interested	22.00% (*n* = 11/50)
	Regularly use AI in research projects	20.00% (*n* = 10/50)
	Plan the future use of AI	18.00% (*n* = 9/50)
	Neither use nor interested in AI	2.00% (*n* = 1/50)

### Structured Programs and Mentorship

Structured research programs for clinical or medical scientists were available at 50.00% (*n* = 21/42) of respondents’ institutions. Among doctoral students, 37.50% (*n* = 15/40) were enrolled in structured programs, while 62.50% (*n* = 25/40) were not. Thematic alignment was common, with 71.43% (*n* = 20/28) of programs focusing on specific research topics. 65.63% (*n* = 21/32) of structured programs were accompanied by mentoring. Outside of structured programs, 43.75% (*n* = 14/32) of researchers reported to having no access to mentoring offers. Satisfaction with mentoring programs was heterogeneous, ranging from very high, such as 100% satisfaction in 8.33% (*n* = 1/12), to low values, such as 20% satisfaction in 16.67% (*n* = 2/12). The positive influence of mentoring programs on research satisfaction stood out: dissatisfaction without mentoring was at 37.50% (*n* = 3/8), compared to only 5.26% (*n* = 1/19) among participants of a mentoring program.

International mobility was limited within the specialty. Only 19.51% (*n* = 8/41) of respondents were able to complete a fellowship abroad. With regard to further research training, 50.00% (*n* = 32/64) of the institutes offered the opportunity to participate in conferences (including leave of absence and cost coverage), followed by seminars 28.13% (*n* = 18/64), workshops 20.13% (*n* = 13/64), and other formats 1.56% (*n* = 1/64) (Table 5).

**Table 5:** Structured Programs and Mentorship

Question	Possible answer	Percent (number/number of respondents to question)
Are clinician or medical scientists enrolled in structured research programs currently working at your institute?	Yes	50.00% (*n* = 21/42)
	No	50.00% (*n* = 21/42)
Do doctoral students enrolled in a structured PhD program work at your institute?	Yes	37.50% (*n* = 15/40)
	No	62.50% (*n* = 25/40)
Are these clinician/medical scientist or PhD programs dedicated to a defined research area?	Yes	71.43% (*n* = 20/28)
	No	28.57% (*n* = 8/28)
Do these programs include structured mentoring?	Yes	65.63% (*n* = 21/32)
	No	34.38% (*n* = 11/32)
If so, how would you rate the quality of the mentoring program? Please rate on a scale from 0 to 100%, where 100% indicates very high satisfaction.	100%	8.33% (*n* = 1/12)
	80%	33.33% (*n* = 4/12)
	60%	16.67% (*n* = 2/12)
	50%	16.67% (*n* = 2/12)
	40%	8.33% (*n* = 1/12)
	20%	16.67% (*n* = 2/12)
Apart from clinician scientist or medical scientist programs, does your institution offer mentoring opportunities for early-career researchers that are open to all members?	No	43.75% (*n* = 14/32)
	Yes	56.25% (*n* = 18/32)
Have you completed a research fellowship abroad?	Yes	19.51% (*n* = 8/41)
	No	80.49% (*n* = 33/41)
What continuing education and training opportunities in research does your institute offer?	Conferences (leave of absence, payment of travel expenses)	50.00% (*n* = 12/33)
	Seminars	28.13% (*n* = 18/33)
	Workshops	20.13% (*n* = 13/33)
	Other	1.56% (*n* = 1/33)

### Funding Sources and Financial Support

The primary funding source was intramural university support 36.76% (*n* = 25/69), followed by the German Research Foundation (DFG) 22.06% (*n* = 15/69) and the Federal Ministry of Education and Research (BMBF) 16.18% (*n* = 11/69). Additional funding came from the Else Kröner-Fresenius Foundation 8.82% (*n* = 6/69), German Cancer Aid, and EU sources 5.88% (*n* = 4/69) each. Few respondents 1.47% (*n* = 1/69) each named the DAAD, Novartis Foundation, Wilhelm Sander Foundation, or international programs.

53.85% (*n* = 21/39) of respondents received industry funding, while 46.15% (*n* = 18/39) worked without such support. Interestingly, publication output was higher among those without industry support: 57.89% (*n* = 11/19) of respondents without industry backing published more than five papers per year, compared to 42.11% (*n* = 8/19) in the industry-funded group. In terms of funding amounts, 36.36% (*n* = 12/33) of respondents received individual grants of up to € 100,000 or up to € 500,000, while 27.27% (*n* = 9/33) reported receiving grants of over € 500,000. Only a few are aware of programs, funding lines, or scholarship programs specifically tailored to neuroradiology. 75.00% (*n* = 18/24) of respondents could not name any such programs (Table 6).

**Table 6:** Funding Sources and Financial Support

Question/Analysis	Possible answer	Percent (number/number of respondents to question)
Who are your primary sources of research funding (multiple answers possible)?	Own university via intramural funding lines	36.76% (*n* = 25/69)
	German Research Foundation (DFG)	22.06% (*n* = 15/69)
	Federal Ministry of Education and Research (BMBF)	16.18% (*n* = 11/69)
	Else Kröner-Fresenius Foundation (EKFS)	8.82% (*n* = 6/69)
	German Cancer Aid	5.88% (*n* = 4/69)
	EU funding	5.88% (*n* = 4/69)
	German Academic Exchange Service (DAAD)	1.47% (*n* = 1/69)
	Novartis Foundation	1.47% (*n* = 1/69)
	Wilhelm Sander Foundation	1.47% (*n* = 1/69)
	International funding programs	1.47% (*n* = 1/69)
Do you receive industry funding for your research work?	Yes	53.85% (*n* = 21/39)
	No	46.15% (*n* = 18/39)
Comparison of the number of original publications per year between researchers with and without industry support	With support from the industry: Original publications < 5 per year	50.00% (*n* = 13/26)
	With support from the industry: Original publications > 5 per year	42.11% (*n* = 8/19)
	Not supported by the industry: Original publications < 5 per year	11.54% (*n* = 3/26)
	Not supported by the industry: Original publications > 5 per year	57.89% (*n* = 11/19)
What funding programs or grants specifically targeted at neuroradiology researchers are you aware of (free text)?	Network of University Medicine (NUM)/BMBF	4.17% (*n* = 1/24)
	Kurt Decker Research award of the DGNR	4.17% (*n* = 1/24)
	Travel grants from the DGNR	4.17% (*n* = 1/24)
	Health.AI	4.17% (*n* = 1/24)
	RACOON – Radiological Cooperative Network/NUM/BMBF	8.33% (*n* = 2/24)
	No scholarships or funding programs known	75.00% (*n* = 18/24)
What was the size of your largest single grant?	Up to 100,000 Euros	36.36% (*n* = 12/33)
	Up to 500,000 Euros	36.36% (*n* = 12/33)
	Over 500,000 Euros	27.27% (*n* = 9/33)

### Publication Activity and Institutional Resources

The analysis of publication performance shows that the respondents’ scientific work appears in both specialty and high-impact journals. Clinical Neuroradiology and the Journal of NeuroInterventional Surgery were each cited as journals of choice for frequent publications by 15.78% (*n* = 6/40) of respondents. Notably, some researchers also published in journals with impact factors (IF) above 7 (e.g., Stroke, Investigative Radiology).

The research-related infrastructure (at least partial research use) varies considerably between the institutes. In terms of MRI equipment, 8 institutes have two devices, 5 have one device, and some have up to five MRIs available for research. The spectrum ranges from specialized research devices such as the 7T Siemens Terra and the preclinical 9.4T Bruker high-field scanners to typically clinically used devices such as 3T scanners. There is also a wide range of equipment in the field of computed tomography (CT) and angiography: e.g. Siemens Naeotom CT, GE Revolution spectral CT, as well as several biplane angiography systems.

Organizational support for academic work varies: 28.20% (*n* = 11/39) of respondents reported IT and administrative support, 25.64% (*n* = 10/39) received research-specific help. 17.94% (*n* = 7/39) stated that they did not receive any institutional support. With regard to statistical consultation, 76.32% (*n* = 29/38) relied on internal expertise, 10.53% (*n* = 4/38) on external expertise, while 13.16% (*n* = 5/38) had no access to such support (Table 7).

**Table 7:** Publication Activity and Institutional Resources

Question/Analysis	Possible answer		Percent (number/number of respondents to question)
In which journal do you publish most frequently? (free text)	Journal of NeuroInterventional Surgery		15.78% (*n* = 6/40)
	Clinical Neuroradiology		15.78% (*n* = 6/40)
	Stroke		7.89% (*n* = 3/40)
	European Radiology		7.89% (*n* = 3/40)
	NeuroImage		5.26% (*n* = 2/40)
	Cerebrovascular Disease		2.63% (*n* = 1/40)
	Neuroradiology		2.63% (*n* = 1/40)
	CardioVascular and Interventional Radiology		2.63% (*n* = 1/40)
	American Journal of Neuroradiology		2.63% (*n* = 1/40)
	Journal of Magnetic Resonance Imaging		2.63% (*n* = 1/40)
	Human Brain Mapping		2.63% (*n* = 1/40)
	Multidisciplinary Digital Publishing Institute		2.63% (*n* = 1/40)
	Neuro-Oncology Advances		2.63% (*n* = 1/40)
	Scientific Reports		2.63% (*n* = 1/40)
	Frontiers in Neurology		2.63% (*n* = 1/40)
	Clinical Nursing Research		2.63% (*n* = 1/40)
	Journal of Neurology		2.63% (*n* = 1/40)
	Investigative Radiology		2.63% (*n* = 1/40)
	Cerebrovascular Disease		2.63% (*n* = 1/40)
	Science Advances		2.63% (*n* = 1/40)
	Theranostics		2.63% (*n* = 1/40)
	BioMed Central		2.63% (*n* = 1/40)
	Journal of Vascular and Interventional Neurology		2.63% (*n* = 1/40)
	Various Springer journals		2.63% (*n* = 1/40)
Relationship between high impact (IF ≥ 7) researchers and their number of publications per year	Up to 5		50.00% (*n* = 3/6)
	Up to 10		33.33% (*n* = 2/6)
	More than 20		16.67% (*n* = 1/6)
What research-related equipment infrastructure is available to you/your working group, either exclusively for research purposes or shared with patient care?	MRI devices	1	19.23% (*n* = 5/26)
		2	30.77% (*n* = 8/26)
		3	11.54% (*n* = 3/26)
		4	11.54% (*n* = 3/26)
		5	3.85% (*n* = 1/26)
	CT devices	1	23.08% (*n* = 6/26)
		2	7.69% (*n* = 2/26)
	Angiography systems	1	23.08% (*n* = 6/26)
		2	15.38% (*n* = 4/26)
	Ultrasound	1	3.85% (*n* = 1/26)
	Additional equipment for research	3	11.54% (*n* = 3/26)
How is the scientific work at your institute supported at the organizational level?	IT support		28.20% (*n* = 11/39)
	Administrative support		28.20% (*n* = 11/39)
	Research-specific support		25.64% (*n* = 10/39)
	None		17.94% (*n* = 7/39)
Do you have access to statistical advice and support?	Have internal		76.32% (*n* = 29/38)
	Have external		10.53% (*n* = 4/38)
	No advice		13.16% (*n* = 5/38)

### Researcher Satisfaction and Outlook

**Fig. 3:**
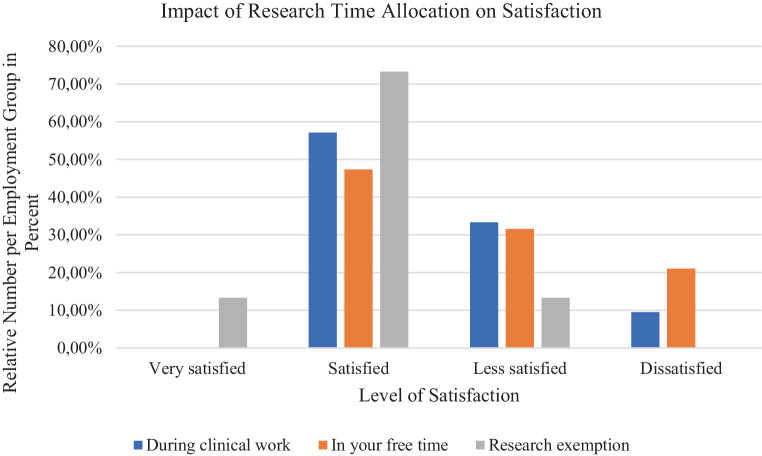
Impact of research time type on satisfaction

Satisfaction levels were strongly associated with access to protected research time. Among those with protected research time, 13.33% (*n* = 2/15) reported being “very satisfied” and 73.33% (*n* = 11/15) “satisfied”. In contrast, with 21.05% (*n* = 4/19), dissatisfaction was highest among those conducting research during their private time (Fig. 3). Group size also influenced satisfaction, with larger groups (> 10 people per group) generally associated with more positive ratings than small (1–5 people per group) or medium-sized groups (6–10 people per group).

The primary barriers to research were lack of time 41.10% (*n* = 30/73), insufficient funding, and limited infrastructure 24.66% (*n* = 18/73) each. Interdisciplinary collaboration was viewed as “very important” by 47.06% (*n* = 16/34) of respondents. When asked about the future of neuroradiology research in Germany, 39.03% (*n* = 16/41) were optimistic, while 21.95% (*n* = 9/41) held a neutral view and 21.95% (*n* = 9/41) expressed concern (negative or very negative) (Table 8).

**Table 8:** Researcher Satisfaction and Outlook

Question/Analysis	Possible answer		Percent (number/number of respondents to question)
Group size related to satisfaction	Very satisfied	1 to ≤ 5 (small)	25.00% (*n* = 2/8)
		> 5 to ≤ 10 (medium)	20.00% (*n* = 2/10)
		> 10 (large)	0.00% (*n* = 0/4)
	Satisfied	1 to ≤ 5 (small)	25.00% (*n* = 2/8)
		> 5 to to ≤ 10 (medium)	40.00% (*n* = 4/10)
		> 10 (large)	100.00% (*n* = 4/4)
	Less satisfied	1 to ≤ 5 (small)	25.00% (*n* = 2/8)
		> 5 to ≤ 10 (medium)	20.00% (*n* = 2/10)
		> 10 (large)	0.00% (*n* = 0/4)
	Dissatisfied	1 to ≤ 5 (small)	25.00% (*n* = 2/8)
		> 5 to ≤ 10 (medium)	20.00% (*n* = 2/10)
		> 10 (large)	0.00% (*n* = 0/4)
What difficulties do you experience in combining clinical and scientific work?	Lack of time		41.10% (*n* = 30/73)
	Lack of financial support		24.66% (*n* = 18/73)
	Lack of infrastructural support		24.66% (*n* = 18/73)
	Other		9.59% (*n* = 7/73)
To what extend does interdisciplinary collaboration contribute to your research activities?	Very large extend		47.06% (*n* = 16/34)
	Large extend		32.35% (*n* = 11/34)
	Medium extend		14.71% (*n* = 5/34)
	Small extend		5.88% (*n* = 2/34)
	No extend		0.00% (*n* = 0/34)
How do you perceive the future of neuroradiology research in Germany?	Very positive		2.44% (*n* = 1/41)
	Positive		36.59% (*n* = 15/41)
	Neutral		21.95% (*n* = 9/41)
	Negative		17.07% (*n* = 7/41)
	Very negative		4.88% (*n* = 2/41)
	Comments on how to improve the future		17.07 (*n* = 7/41)
How satisfied are you with the current research opportunities at your institute?	Very satisfied		6.06% (*n* = 2/33)
	Satisfied		48.48% (*n* = 16/33)
	Less satisfied		33.33% (*n* = 11/33)
	Dissatisfied		12.12% (*n* = 4/33)
Impact of protected research time on researchers’ satisfaction	Very satisfied	During working hours in addition to clinical work	0.00% (*n* = 0/21)
		In your free time	0.00% (*n* = 0/19)
		Research exemption	13.33% (*n* = 2/15)
	Satisfied	During working hours in addition to clinical work	57.14% (*n* = 12/21)
		In your free time	47.37% (*n* = 9/19)
		Research exemption	73.33% (*n* = 11/15)
	Less satisfied	During working hours in addition to clinical work	33.33% (*n* = 7/21)
		In your free time	31.58% (*n* = 6/19)
		Research exemption	13.33% (*n* = 2/15)
	Dissatisfied	During working hours in addition to clinical work	9.52% (*n* = 2/21)
		In your free time	21.05% (*n* = 4/19)
		Research exemption	0.00% (*n* = 0/15)
Influence of a mentoring program on researchers’ satisfaction	Very satisfied	Available	10.53% (*n* = 2/19)
		Not available	0.00% (*n* = 0/8)
	Satisfied	Available	68.42% (*n* = 13/19)
		Not available	25.00% (*n* = 2/8)
	Less satisfied	Available	15.79% (*n* = 3/19)
		Not available	37.50% (*n* = 3/8)
	Dissatisfied	Available	5.26% (*n* = 1/19)
		Not available	37.50% (*n* = 3/8)

## Discussion

This national survey provides an overview of the research environment in German neuroradiology, focusing on structural organization, research time allocation, funding, mentoring, and researcher satisfaction. Across these domains, three themes consistently emerged: protected research time remains scarce; institutional support and research infrastructure are varied; and discipline-specific funding schemes and mentoring opportunities are limited. The following sections outline how these factors jointly shape research opportunities and job satisfaction in neuroradiology and discuss the resulting implications for academic policy and departmental leadership.

### Participant Characteristics and Staffing

The DGNR survey provides a snapshot of the current research landscape for neuroradiology professionals in Germany and illustrates how institutional structures and staffing patterns frame research activity. In total, 60 neuroradiologists from across the federal states—excluding Berlin, Brandenburg, and Bremen—partook in the survey; 33 completed it in full and 27 discontinued partway. Thus, the effective sample size for most analyses was limited (*n* = 33), and all results should be interpreted descriptively, as the sample remains small and is not representative of all neuroradiologists in Germany.

### Institutional Structure

One key insight concerns institutional structure: 69.23% of respondents indicated that they work within independent neuroradiology institutes. This reflects a growing trend towards structural independence in the field—a development aligned with increasing subspecialization in medicine and rising expectations in research and education. As in other medical disciplines, specialization is a prerequisite for scientific excellence [12].

Respondents from independent institutes generally reported higher job satisfaction. While the overall sentiment was positive, some still indicated only moderate satisfaction, suggesting room for improvement. By contrast, respondents from non-independent departments expressed more dissatisfaction, with no one reporting being “very satisfied” and nearly half expressing dissatisfaction. This disparity points to potential structural disadvantages such as reduced autonomy or limited resources. This pattern parallels developments in other specialties like rheumatology, where independence within university structures is also seen as crucial for fulfilling teaching obligations and ensuring high-quality training [13].

### Research Focus and Time Allocation

As in many medical disciplines, neuroradiology is confronted with the ongoing challenge of combining research activities with clinical responsibilities and private life. In this survey, time allocation appears as a central determinant of whether the existing structural framework can be translated into effective research output. According to survey data, 39.39% of research in neuroradiology is primarily performed during regular working hours alongside clinical duties, suggesting a degree of institutional integration between clinical and scientific tasks. Nevertheless, 37.88% of respondents primarily conduct research during their private time, indicating both a high clinical workload and significant intrinsic motivation. Compared to other specialties, this is a more favourable distribution: in rheumatology, 71.6% [10]; in urology, 48% [14]; and in radiology, as high as 87% of research is conducted during personal time [2].

A further concern is the discrepancy between formally allocated and actually available research time. This gap may result from organizational barriers that prevent researchers from making full use of protected time, thereby complicating the systematic planning and execution of research projects. The particular nature of neuroradiology further intensifies this challenge: a significant share of clinical practice is devoted to emergency medicine, which requires continuous flexibility and rapid response from physicians [15]. Consequently, safeguarding dedicated research time is especially difficult in this field, particularly given the comparatively limited personnel resources.

Empirical evidence highlights the extent of this problem: between 2009 and 2023, the workload in acute neuroradiology during on-call hours increased by approximately 130%, while staffing levels per shift remained unchanged. In particular, the number of MRI examinations rose substantially during this period, indicating a significant increase in clinical demand [16]. Although multiple factors may contribute to this development, the key implication is a marked intensification of workload, leaving less time and cognitive capacity available for research.

### Use of Artificial Intelligence in Research

Currently, clinical research in neuroradiology is focused primarily on neurovascular topics (27%) and cross-sectional neuroimaging (25%)—the latter being crucial for diagnosing cerebrovascular diseases, cancer, neurodegeneration, trauma, and neuroinflammation [17]. However, future research directions remain uncertain. Artificial intelligence (AI) has emerged as a promising area. While its use is currently project-based and situational, nearly all respondents view AI as a highly relevant technology and express a strong interest in exploring it further. This reflects the increasing importance of AI in the field. Initial fears that AI might replace radiologists have since evolved [18, 19]. Much like pilots are still essential in aviation despite autopilot systems, radiologists remain indispensable, particularly for complex decision-making. As C. Langlotz stated, the critical question is not whether AI will replace radiologists, but whether radiologists who utilize AI will replace those who do not [18].

### Structured Programs

Structured research programs at university hospitals play a crucial role in advancing scientific careers. As institutions mandated to conduct research, these hospitals are essential in translating scientific knowledge into clinical practice [20]. Interdisciplinary collaboration and targeted support for early-career researchers are fundamental and often facilitated by structured programs [21]. Such programs are currently established in about 50% of the respondents’ institutes, a figure comparable to surveys conducted among general radiologists (52%) [2], urologist (30%) [11], and rheumatologists (22.5%) [10]. A lack of structured support is often cited as a major cause of dissatisfaction [11]. Data from the BIH Charité Clinician Scientist Program indicate that well-structured research environments correlate with a lower perceived workload [22].

### Mentorship

Mentoring programs show particularly strong impact: 65.63% of structured research programs include mentoring components. Notably, researchers with access to mentoring reported higher satisfaction levels, with only mentees reaching the highest satisfaction category. Effective mentoring is not only key to personal and professional development but is also associated with increased publication rates and greater career advancement [23].

However, mentoring must be carefully managed. Previous studies found that 30% of women and 10% of men felt their mentors primarily used their work to advance their own careers [24, 25]. Thus, mentoring relationships should be clearly defined and regularly evaluated. According to Straus et al., good mentoring is rooted in altruism [26] and characterized by active listening, honesty, and trust [26–30]. The DFG also explicitly recommends formal mentoring structures for sustainable academic development [20].

To foster this sentiment, the NUNRAD Network of the German Society of Neuroradiology (DGNR) serves as a platform for university representatives in neuroradiology, independent of academic title. Its objectives are to promote communication within and beyond the discipline and to strengthen its visibility in scientific and science policy contexts [31]. From this initiative, the first multicenter project has emerged: RACOON-AI Brain Tumor, a nationwide collaborative effort in neuro-oncology. The project focuses on advanced imaging techniques to improve diagnosis, treatment planning, and patient outcomes in brain tumors. It is based on close collaboration among neuroradiologists, neuro-oncologists, neurosurgeons, data scientists, and patient advocates. Led by an interdisciplinary team, the project is supported by both the DGNR and NUNRAD [32].

### Funding Sources and Financial Support

Currently, research in neuroradiology is primarily funded through intramural university resources (36.76%) and third-party grants from public organizations such as the DFG and the BMBF. Dedicated scholarship and funding programs remain scarce−75% of respondents were unaware of any specific offers in the field. This reflects a lack of visibility and availability of discipline-specific funding.

To address this, the creation of centralized information resources—such as a digital platform summarizing available funding options—would be highly beneficial. Additionally, expanding targeted, subject-specific funding lines is crucial. A positive step in this direction is the DGNR’s 2024 launch of a travel grant program, set to support conference and workshop participation from 2025 onward [33]. Networks such as NUNRAD can strengthen the funding landscape indirectly by creating visibility and facilitating large-scale collaborative projects like RACOON-AI Brain Tumor, which in turn could attract external support [32].

### Publication Activity and Institutional Resources

Publication activity among German university-based neuroradiologists primarily targets specialist journals like Clinical Neuroradiology (IF: 2.6 (2024)) and the Journal of NeuroInterventional Surgery. Publications in high-impact interdisciplinary journals (IF > 7), such as Stroke, Investigative Radiology, or Science Advances, are rare and largely limited to a few individuals, with one exceptionally prolific researcher producing over 20 articles annually.

Although IF remains a key metric in evaluating scientific performance and allocating funding, it is not without criticism. Field-specific publication patterns and issues such as self-citation can distort evaluations [34, 35]. In highly specialized disciplines like neuroradiology, a lower IF often reflects structural characteristics rather than scientific quality [35, 36]. A differentiated assessment of IF is therefore essential [35].

The institutes provide organizational support for scientific work at various levels: 28% of respondents report access to IT and administrative support, 26% receive research-specific assistance, and 18% report receiving no support at all—highlighting a heterogeneous research infrastructure across institutes.

### Researcher Satisfaction and Outlook

Consistent with these structural and resource-related findings, researcher satisfaction in this survey is closely linked to the availability of protected research time, institutional support, and group size. Taken together, these results suggest that improving research conditions in neuroradiology requires coordinated action at both the institutional and systemic level. Those with protected time reported higher satisfaction: 13.33% were “very satisfied” and 73.33% “satisfied”. By contrast, those conducting research in their free time expressed higher dissatisfaction with 21.05%, and with none in this group reporting high satisfaction. These findings underscore the importance of structured, protected research time for both productivity and work-life balance. In accordance, clinician scientists with a 50:50 split between protected research time and patient care were the most satisfied within the BIH Charité (J)CSP [22].

The dual demands of clinical and scientific work remain a major challenge. Time constraints are the most frequently cited barrier, followed by inadequate financial and infrastructure support— challenges common to other medical fields such as rheumatology [10], urology [11], and radiology [2]. An increasingly popular solution is the implementation of specific personnel-based funding models, such as clinician scientist programs or other rotation models that finance qualified clinical staff to temporarily replace clinical researchers ensuring protected research time without compromising patient care [37]. Additionally, a stronger emphasis on third-party funding could improve institutional resources.

Notably, 79.04% of respondents emphasized the importance of interdisciplinary collaboration. This highlights the need to foster interdisciplinary structures through joint research projects, networks, and targeted funding [37, 38]. Group size also affects satisfaction: larger research groups tend to correlate with higher satisfaction levels due to greater resources. However, this is not universal—some researchers in large groups reported dissatisfaction, potentially due to limited individual attention or difficulty establishing academic independence.

Despite these challenges, overall sentiment remains optimistic. Most researchers assess their current research opportunities positively and are hopeful about the future of neuroradiology research. This reflects a strong identification with the field and high intrinsic motivation—key elements for sustained academic success.

## Limitations

The study is subject to several potential sources of bias, including selection and interest bias, the relatively small number of complete responses (*n* = 33), leadership bias due to the high proportion of respondents in senior positions, social desirability bias in self-reported assessments, and attrition bias related to the high dropout rate with 27 out of 60 respondents providing only partial data. Based on membership figures from the DGNR and its junior section JuNRAD, an estimated 500–900 neuroradiologists in Germany are regularly involved in research [39]. Against this background, the 33 complete responses correspond to an estimated response proportion of about 4–7%, which is lower than response rates reported in many web-based surveys of physicians and medical specialists [40]. Consequently, the findings should be interpreted as exploratory and cannot be regarded as statistically representative of all German neuroradiologists.

Young researchers appeared to be underrepresented in the sample. Despite the additional use of social media channels to reach younger researchers, their responses remained relatively scarce. This likely biased the results toward more senior, established researchers and institutional leaders and may have led to an underestimation of challenges and support needs specific to early-career neuroradiologists. In addition, subgroup sizes (e.g. for doctoral students and postdocs) were very small; subgroup analyses are therefore purely descriptive and cannot support inferential conclusions. Moreover, individual demographic variables such as age and sex were not collected, which precludes the analysis of sex- or age-specific differences and limits comparability with other survey data.

The comprehensive scope and cognitive load of the 33-item questionnaire likely contributed to the high dropout rate and reduced the feasibility of cross-analyses between early and later questions. Limited perceived personal benefit or incentives, competing time demands among clinically and academically active respondents, and the inclusion of more personal or evaluative items (e.g. regarding satisfaction) may have further discouraged completion and contributed to selective participation. In some cases, despite piloting the survey, comprehension issues led to contradictory responses. Additionally, identifying current and accurate email addresses proved challenging; many university websites were outdated, making it unclear whether the intended target group of researchers was consistently reached. Extending the survey period might have improved response rates and expanded the data set. Moreover, no specific methodological measures were implemented to mitigate these biases; they should therefore be considered carefully when interpreting the findings. Finally, whether research conditions have changed since the survey period is unknown, although no nationwide structural reforms have occurred that would indicate major changes in the general framework.

## Conclusion

Taken together, these findings outline a consistent picture of the neuroradiology research ecosystem in Germany: many departments have established independent structures and active research groups, yet limited protected research time, insufficient financial and infrastructural support, and limited structured research programs and mentorship constrain the full realisation of this potential. At the same time, emerging trends such as the rise of AI, growing interdisciplinary collaboration, and the increasing professionalisation of research careers offer substantial opportunities if accompanied by targeted funding, protected leave models, adequate personnel resources, and formal mentoring programmes. The strong intrinsic motivation and optimism within the neuroradiology research community provide a solid foundation for further developing the field into an innovative and research-intensive medical specialty.
